# *trans*-Diaqua­bis­[5-carb­oxy-4-carboxyl­ato-2-(4-pyridinio)-1*H*-imidazol-1-ido-κ^2^ *N* ^3^,*O* ^4^]zinc(II)

**DOI:** 10.1107/S1600536810031855

**Published:** 2010-08-28

**Authors:** Xia Li, Ling-Zhi Du, Ben-Lai Wu, Hong-Yun Zhang

**Affiliations:** a Department of Chemistry and Chemical Engineering, Henan University of Urban Construction, Pingdingshan, Henan 467044, People’s Republic of China; b Department of Chemistry, Zhengzhou University, Zhengzhou, Henan 450052, People’s Republic of China

## Abstract

In the title complex, [Zn(C_10_H_6_N_3_O_4_)_2_(H_2_O)_2_], the Zn^II^ atom is located on a twofold rotation axis and is coordinated by two *trans*-positioned *N*,*O*-bidentate and zwitterionic 5-carb­oxy-4-carboxyl­ato-2-(4-pyridinio)-1*H*-imidazol-1-ide (H_2_PIDC^−^) ligands and two water mol­ecules, defining a distorted octa­hedral environment. The complete solid-state structure can be described as a three-dimensional supra­molecular framework, stabilized by extensive hydrogen-bonding inter­actions involving the coordinated water mol­ecules, uncoordin­ated imidazole N atom, protonated pyridine N and carboxyl­ate O atoms of the H_2_PIDC^−^ ligands.

## Related literature

For related structures, see: Li, Liu *et al.* (2009 ▶); Li, Wu *et al.* (2009 ▶). For the preparation of 2-(pyridin-4-yl)-1*H*-imidazole-4,5-dicarb­oxy­lic acid, see: Sun *et al.* (2006 ▶).
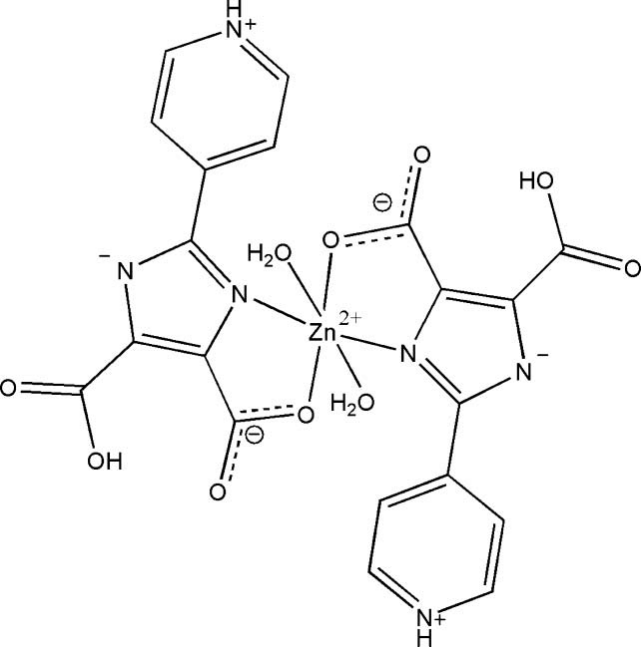


## Experimental

### 

#### Crystal data

[Zn(C_10_H_6_N_3_O_4_)_2_(H_2_O)_2_]
*M* *_r_* = 565.76
Monoclinic, 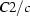

*a* = 7.4138 (9) Å
*b* = 20.204 (3) Å
*c* = 13.4778 (17) Å
β = 97.008 (1)°
*V* = 2003.7 (4) Å^3^
*Z* = 4
Mo *K*α radiation
μ = 1.31 mm^−1^
*T* = 173 K
0.27 × 0.17 × 0.10 mm

#### Data collection

Rigaku Mercury CCD diffractometer
Absorption correction: multi-scan (*CrystalClear*; Rigaku, 2000 ▶) *T*_min_ = 0.754, *T*_max_ = 0.878
9235 measured reflections
2488 independent reflections
1957 reflections with *I* > 2σ(*I*)
*R*_int_ = 0.031

#### Refinement

*R*[*F* ^2^ > 2σ(*F* ^2^)] = 0.031
*wR*(*F* ^2^) = 0.090
*S* = 1.04
2488 reflections
178 parameters
1 restraint
H atoms treated by a mixture of independent and constrained refinement
Δρ_max_ = 0.34 e Å^−3^
Δρ_min_ = −0.37 e Å^−3^

### 

Data collection: *CrystalClear* (Rigaku, 2000 ▶); cell refinement: *CrystalClear*; data reduction: *CrystalClear*; program(s) used to solve structure: *SHELXS97* (Sheldrick, 2008 ▶); program(s) used to refine structure: *SHELXL97* (Sheldrick, 2008 ▶); molecular graphics: *SHELXTL* (Sheldrick, 2008 ▶); software used to prepare material for publication: *SHELXTL*.

## Supplementary Material

Crystal structure: contains datablocks I, global. DOI: 10.1107/S1600536810031855/jh2196sup1.cif

Structure factors: contains datablocks I. DOI: 10.1107/S1600536810031855/jh2196Isup2.hkl

Additional supplementary materials:  crystallographic information; 3D view; checkCIF report

## Figures and Tables

**Table 1:** Selected bond lengths (Å)

Zn1—O2	2.0713 (15)
Zn1—O1	2.1407 (18)
Zn1—N1	2.1592 (17)

**Table 2:** Hydrogen-bond geometry (Å, °)

*D*—H⋯*A*	*D*—H	H⋯*A*	*D*⋯*A*	*D*—H⋯*A*
O1—H1*B*⋯N2^i^	0.82 (3)	2.08 (3)	2.898 (3)	178 (3)
N3—H3⋯O5^ii^	0.88	1.89	2.755 (2)	169
O4—H4*A*⋯O3	0.89 (2)	1.58 (2)	2.459 (2)	173 (3)

Symmetry codes: (i) 
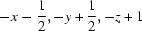
; (ii) 
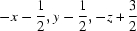
.

**Fig. 1.**
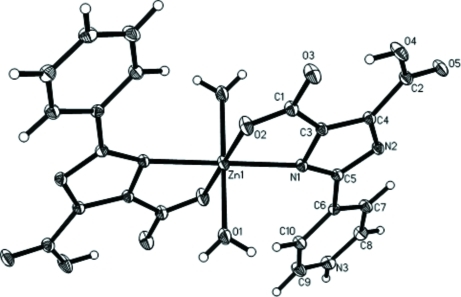
A view of the molecular of (I), showing the atom-labelling scheme and displacement ellipsoids at the 30% probability level.

**Fig. 2.**
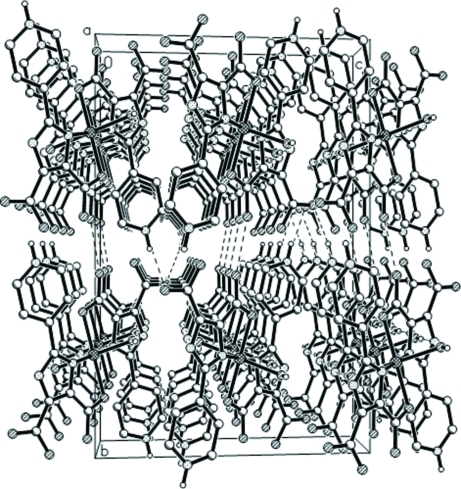
The crystal packing of (I), showing the three-dimensional hydrogen-bonding network, H atoms have been omited for clarity.

## supplementary crystallographic information

### Comment

Multifunctional connector 2-(pyridin-4-yl)-1*H*-imidazole-4,5-dicarboxylate acid (H_3_PIDC), a rigid N-heterocyclic carboxylate, has great potential for coordinative interactions and hydrogen bonding, showing more interesting traits in the construction of MOFs. It can be successively deprotonated to generate various species with different proton numbers, and hence may result in a large diversity of supramolecular architectures. Very recently, we have reported several supramolecular architectures (Li, Wu *et al.*, 2009; Li, Liu *et al.*, 2009) base on ligand 2-(pyridin-4-yl)-1*H*-imidazole-4,5-dicarboxylic acid. As an extension of our previous investigations, we have isolated a new Zn(II) complex, [Zn(H_2_PIDC)_2_(H_2_O)_2_], (I), by the reaction of H_3_PIDC and Zn(II) diacetate under the hydrothermal condition. We report here the single-crystal structure of this complex.

As shown in Fig. 1, the molecule of (I) is a discrete neutral monomer, in which the Zn atom resides on a crystallographic inversion centre and the asymmetric unit contains one-half of the [Zn(H_2_PIDC)_2_(H_2_O)_2_] formula unit. Each Zn atom is six-coordinated by N_2_O_4_ with two chelating rings from two H_2_PIDC ligands arranged symmetrically in the equatorial plane and two water molecules occupying the apical sites, showing a distorted octahedral geometry (Table 1). In this complex, one carboxyl group and imidazole group are deprotonated and the pyridyl group is protonated, and the ligand bears a formal charge of -1, and the uncoordinated carboxylate atoms O3 and O4 form an intramolecular hydrogen bond (Table 2). All non-H atoms in the imidazole-4,5-dicarboxyl group are nearly coplanar [the mean deviation is 0.075 (3) Å], and the dihedral angle between imidazole group and pyridine group is 11.4 (2) °.

A three-dimensional supramolecular network is constructed *via* hydrogen-bonding interactions involving the coordinated water molecules, uncoordinated imidazole N atom, protonated pyridine N and carboxylate O atoms of the H_2_PIDC^-^ ligands (Table 2 and Fig. 2).

### Experimental

A mixture of zinc diacetate dihydrate (0.022 g, 0.1 mmol), 2-(pyridin-4-yl)-1*H*-imidazole-4,5-dicarboxylic acid (0.024 g, 0.1 mmol) (Sun *et al.*, 2006), NaOH (0.004 g, 0.1 mmol) and water (10 ml) was sealed into a Teflon-lined stainless autoclave and heated at 413 K for 3 days, then cooled to room temperature gradually and colourless block crystals of (I) were obtained. Analysis calculated for C_20_H_16_ZnN_6_O_10_: C 42.46, H 2.85, N 14.85; found: C 42.82, H 2.73, N 14.70.

### Refinement

H atoms attached to N and O atoms were located in a difference Fourier maps and refined as riding in their as-found relative positions, with *U*_iso_(H) = 1.5*U*_eq_(O,*N*). Other H atoms were positioned geometrically with C—H = 0.95 Å and constrained to ride on their parent atoms with *U*_iso_(H) = 1.2*U*_eq_(C).

### Crystal data

**Table:**

[Zn(C_10_H_6_N_3_O_4_)_2_(H_2_O)_2_]	*F*(000) = 1152
*M**_r_* = 565.76	*D*_x_ = 1.875 Mg m^−^^3^
Monoclinic, *C*2/*c*	Mo *K*α radiation, λ = 0.71073 Å
*a* = 7.4138 (9) Å	θ = 2.5–28.3°
*b* = 20.204 (3) Å	µ = 1.31 mm^−^^1^
*c* = 13.4778 (17) Å	*T* = 173 K
β = 97.008 (1)°	Block, colorless
*V* = 2003.7 (4) Å^3^	0.27 × 0.17 × 0.10 mm
*Z* = 4	

### Data collection

**Table:**

Rigaku Mercury CCD diffractometer	2488 independent reflections
Radiation source: fine-focus sealed tube	1957 reflections with *I* > 2σ(*I*)
graphite	*R*_int_ = 0.031
ω scan	θ_max_ = 28.3°, θ_min_ = 2.5°
Absorption correction: multi-scan (*CrystalClear*; Rigaku, 2000)	*h* = −9→9
*T*_min_ = 0.754, *T*_max_ = 0.878	*k* = −26→26
9235 measured reflections	*l* = −17→17

### Refinement

**Table:**

Refinement on *F*^2^	Primary atom site location: structure-invariant direct methods
Least-squares matrix: full	Secondary atom site location: difference Fourier map
*R*[*F*^2^ > 2σ(*F*^2^)] = 0.031	Hydrogen site location: inferred from neighbouring sites
*wR*(*F*^2^) = 0.090	H atoms treated by a mixture of independent and constrained refinement
*S* = 1.04	*w* = 1/[σ^2^(*F*_o_^2^) + (0.0453*P*)^2^ + 2.1588*P*] where *P* = (*F*_o_^2^ + 2*F*_c_^2^)/3
2488 reflections	(Δ/σ)_max_ < 0.001
178 parameters	Δρ_max_ = 0.34 e Å^−^^3^
1 restraint	Δρ_min_ = −0.37 e Å^−^^3^

### Special details

**Table:**

Geometry. All e.s.d.'s (except the e.s.d. in the dihedral angle between two l.s. planes) are estimated using the full covariance matrix. The cell e.s.d.'s are taken into account individually in the estimation of e.s.d.'s in distances, angles and torsion angles; correlations between e.s.d.'s in cell parameters are only used when they are defined by crystal symmetry. An approximate (isotropic) treatment of cell e.s.d.'s is used for estimating e.s.d.'s involving l.s. planes.
Refinement. Refinement of *F*^2^ against ALL reflections. The weighted *R*-factor *wR* and goodness of fit *S* are based on *F*^2^, conventional *R*-factors *R* are based on *F*, with *F* set to zero for negative *F*^2^. The threshold expression of *F*^2^ > σ(*F*^2^) is used only for calculating *R*-factors(gt) *etc*. and is not relevant to the choice of reflections for refinement. *R*-factors based on *F*^2^ are statistically about twice as large as those based on *F*, and *R*- factors based on ALL data will be even larger.

### Fractional atomic coordinates and isotropic or equivalent isotropic displacement parameters (Å^2^)

**Table:**

	*x*	*y*	*z*	*U*_iso_*/*U*_eq_	
Zn1	0.2500	0.2500	0.5000	0.02158 (12)	
O1	0.1031 (3)	0.21527 (11)	0.36331 (14)	0.0405 (5)	
N1	−0.0003 (2)	0.26283 (8)	0.56460 (13)	0.0181 (3)	
C1	0.0872 (3)	0.37351 (10)	0.51444 (16)	0.0230 (4)	
O2	0.1956 (2)	0.34788 (7)	0.46161 (13)	0.0296 (4)	
N2	−0.2095 (2)	0.28420 (8)	0.67035 (13)	0.0204 (4)	
C2	−0.2007 (3)	0.40644 (10)	0.68011 (17)	0.0239 (4)	
O3	0.0612 (2)	0.43586 (7)	0.51721 (13)	0.0355 (4)	
N3	−0.2067 (3)	0.03644 (9)	0.68078 (15)	0.0283 (4)	
H3	−0.2203	−0.0056	0.6953	0.034*	
C3	−0.0184 (3)	0.32960 (9)	0.57348 (15)	0.0184 (4)	
O4	−0.1528 (3)	0.46001 (7)	0.63823 (14)	0.0368 (4)	
C4	−0.1451 (3)	0.34251 (9)	0.63948 (15)	0.0192 (4)	
O5	−0.2869 (2)	0.40756 (8)	0.75300 (12)	0.0322 (4)	
C5	−0.1194 (3)	0.23786 (9)	0.62332 (15)	0.0184 (4)	
C6	−0.1542 (3)	0.16745 (9)	0.63892 (15)	0.0180 (4)	
C7	−0.2469 (3)	0.14843 (10)	0.71861 (17)	0.0250 (5)	
H7	−0.2949	0.1811	0.7587	0.030*	
C8	−0.2687 (3)	0.08256 (11)	0.73901 (17)	0.0278 (5)	
H8	−0.3279	0.0698	0.7946	0.033*	
C9	−0.1247 (3)	0.05237 (11)	0.60116 (18)	0.0311 (5)	
H9	−0.0866	0.0184	0.5597	0.037*	
C10	−0.0947 (3)	0.11772 (10)	0.57857 (17)	0.0251 (5)	
H10	−0.0343	0.1288	0.5226	0.030*	
H1B	−0.008 (4)	0.2148 (13)	0.353 (2)	0.030*	
H1A	0.135 (4)	0.1903 (13)	0.324 (2)	0.030*	
H4A	−0.080 (3)	0.4538 (12)	0.5916 (16)	0.030*	

### Atomic displacement parameters (Å^2^)

**Table:**

	*U*^11^	*U*^22^	*U*^33^	*U*^12^	*U*^13^	*U*^23^
Zn1	0.0248 (2)	0.01734 (18)	0.0245 (2)	0.00358 (13)	0.01053 (13)	0.00102 (13)
O1	0.0245 (9)	0.0669 (14)	0.0305 (10)	0.0044 (9)	0.0054 (8)	−0.0200 (9)
N1	0.0208 (8)	0.0139 (8)	0.0206 (8)	0.0008 (6)	0.0066 (7)	0.0011 (6)
C1	0.0266 (11)	0.0175 (10)	0.0261 (11)	0.0015 (8)	0.0088 (9)	0.0026 (8)
O2	0.0361 (9)	0.0201 (7)	0.0366 (9)	0.0046 (7)	0.0203 (7)	0.0067 (7)
N2	0.0216 (9)	0.0161 (8)	0.0250 (9)	−0.0002 (7)	0.0085 (7)	−0.0004 (7)
C2	0.0263 (11)	0.0184 (10)	0.0280 (11)	0.0016 (8)	0.0077 (9)	−0.0012 (8)
O3	0.0493 (11)	0.0144 (7)	0.0482 (11)	0.0022 (7)	0.0275 (9)	0.0071 (7)
N3	0.0356 (11)	0.0140 (8)	0.0359 (11)	−0.0042 (7)	0.0074 (8)	0.0035 (7)
C3	0.0209 (10)	0.0140 (9)	0.0212 (10)	0.0006 (7)	0.0063 (8)	0.0004 (7)
O4	0.0576 (12)	0.0155 (7)	0.0430 (10)	0.0036 (7)	0.0291 (9)	−0.0001 (7)
C4	0.0222 (10)	0.0144 (9)	0.0220 (10)	0.0007 (7)	0.0062 (8)	0.0013 (7)
O5	0.0417 (10)	0.0220 (8)	0.0370 (9)	0.0018 (7)	0.0215 (8)	−0.0053 (7)
C5	0.0204 (10)	0.0153 (9)	0.0199 (10)	−0.0008 (7)	0.0045 (8)	0.0015 (7)
C6	0.0186 (10)	0.0145 (9)	0.0213 (10)	−0.0013 (7)	0.0034 (8)	0.0016 (7)
C7	0.0309 (12)	0.0183 (10)	0.0275 (11)	−0.0017 (8)	0.0101 (9)	−0.0008 (8)
C8	0.0324 (12)	0.0239 (11)	0.0287 (12)	−0.0055 (9)	0.0109 (10)	0.0054 (9)
C9	0.0411 (14)	0.0190 (10)	0.0353 (13)	−0.0016 (9)	0.0137 (10)	−0.0037 (9)
C10	0.0312 (12)	0.0195 (10)	0.0266 (11)	−0.0023 (8)	0.0115 (9)	−0.0011 (8)

### Geometric parameters (Å, °)

**Table:**

Zn1—O2	2.0713 (15)	C2—O4	1.290 (3)
Zn1—O2^i^	2.0713 (15)	C2—C4	1.481 (3)
Zn1—O1	2.1407 (18)	N3—C9	1.336 (3)
Zn1—O1^i^	2.1407 (18)	N3—C8	1.335 (3)
Zn1—N1^i^	2.1592 (17)	N3—H3	0.8800
Zn1—N1	2.1592 (17)	C3—C4	1.395 (3)
O1—H1B	0.82 (3)	O4—H4A	0.885 (16)
O1—H1A	0.78 (3)	C5—C6	1.466 (2)
N1—C5	1.353 (3)	C6—C7	1.398 (3)
N1—C3	1.362 (2)	C6—C10	1.397 (3)
C1—O2	1.249 (3)	C7—C8	1.372 (3)
C1—O3	1.276 (2)	C7—H7	0.9500
C1—C3	1.478 (3)	C8—H8	0.9500
N2—C5	1.352 (3)	C9—C10	1.379 (3)
N2—C4	1.355 (2)	C9—H9	0.9500
C2—O5	1.236 (3)	C10—H10	0.9500
			
O2—Zn1—O2^i^	180.0	C9—N3—C8	121.80 (19)
O2—Zn1—O1	92.00 (8)	C9—N3—H3	119.1
O2^i^—Zn1—O1	88.00 (8)	C8—N3—H3	119.1
O2—Zn1—O1^i^	88.00 (8)	N1—C3—C4	108.82 (17)
O2^i^—Zn1—O1^i^	92.00 (8)	N1—C3—C1	118.85 (17)
O1—Zn1—O1^i^	180.0	C4—C3—C1	132.32 (18)
O2—Zn1—N1^i^	99.47 (6)	C2—O4—H4A	114.6 (16)
O2^i^—Zn1—N1^i^	80.53 (6)	N2—C4—C3	108.85 (17)
O1—Zn1—N1^i^	89.17 (7)	N2—C4—C2	121.34 (18)
O1^i^—Zn1—N1^i^	90.83 (7)	C3—C4—C2	129.67 (18)
O2—Zn1—N1	80.53 (6)	N1—C5—N2	114.27 (17)
O2^i^—Zn1—N1	99.47 (6)	N1—C5—C6	125.79 (18)
O1—Zn1—N1	90.83 (7)	N2—C5—C6	119.94 (18)
O1^i^—Zn1—N1	89.17 (7)	C7—C6—C10	117.99 (18)
N1^i^—Zn1—N1	180.0	C7—C6—C5	119.26 (18)
Zn1—O1—H1B	123.2 (19)	C10—C6—C5	122.73 (18)
Zn1—O1—H1A	129 (2)	C8—C7—C6	120.1 (2)
H1B—O1—H1A	105 (3)	C8—C7—H7	120.0
C5—N1—C3	103.85 (16)	C6—C7—H7	120.0
C5—N1—Zn1	147.69 (13)	N3—C8—C7	120.1 (2)
C3—N1—Zn1	104.67 (12)	N3—C8—H8	119.9
O2—C1—O3	122.40 (19)	C7—C8—H8	119.9
O2—C1—C3	118.55 (18)	N3—C9—C10	120.6 (2)
O3—C1—C3	119.02 (18)	N3—C9—H9	119.7
C1—O2—Zn1	111.87 (13)	C10—C9—H9	119.7
C5—N2—C4	104.20 (17)	C9—C10—C6	119.3 (2)
O5—C2—O4	121.93 (19)	C9—C10—H10	120.4
O5—C2—C4	120.28 (19)	C6—C10—H10	120.4
O4—C2—C4	117.77 (19)		
			
O2—Zn1—N1—C5	−170.7 (3)	N1—C3—C4—N2	−1.3 (2)
O2^i^—Zn1—N1—C5	9.3 (3)	C1—C3—C4—N2	177.0 (2)
O1—Zn1—N1—C5	97.4 (3)	N1—C3—C4—C2	174.4 (2)
O1^i^—Zn1—N1—C5	−82.6 (3)	C1—C3—C4—C2	−7.3 (4)
N1^i^—Zn1—N1—C5	37 (16)	O5—C2—C4—N2	10.2 (3)
O2—Zn1—N1—C3	−19.40 (13)	O4—C2—C4—N2	−171.3 (2)
O2^i^—Zn1—N1—C3	160.60 (13)	O5—C2—C4—C3	−165.1 (2)
O1—Zn1—N1—C3	−111.28 (14)	O4—C2—C4—C3	13.5 (4)
O1^i^—Zn1—N1—C3	68.72 (14)	C3—N1—C5—N2	−1.2 (2)
N1^i^—Zn1—N1—C3	−171 (100)	Zn1—N1—C5—N2	150.2 (2)
O3—C1—O2—Zn1	166.35 (18)	C3—N1—C5—C6	179.3 (2)
C3—C1—O2—Zn1	−15.4 (3)	Zn1—N1—C5—C6	−29.3 (4)
O2^i^—Zn1—O2—C1	24 (100)	C4—N2—C5—N1	0.4 (2)
O1—Zn1—O2—C1	110.06 (17)	C4—N2—C5—C6	179.97 (19)
O1^i^—Zn1—O2—C1	−69.94 (17)	N1—C5—C6—C7	164.9 (2)
N1^i^—Zn1—O2—C1	−160.45 (16)	N2—C5—C6—C7	−14.7 (3)
N1—Zn1—O2—C1	19.55 (16)	N1—C5—C6—C10	−13.6 (3)
C5—N1—C3—C4	1.5 (2)	N2—C5—C6—C10	166.9 (2)
Zn1—N1—C3—C4	−163.20 (14)	C10—C6—C7—C8	3.5 (3)
C5—N1—C3—C1	−177.10 (19)	C5—C6—C7—C8	−175.1 (2)
Zn1—N1—C3—C1	18.2 (2)	C9—N3—C8—C7	−0.8 (4)
O2—C1—C3—N1	−3.1 (3)	C6—C7—C8—N3	−2.2 (3)
O3—C1—C3—N1	175.2 (2)	C8—N3—C9—C10	2.6 (4)
O2—C1—C3—C4	178.8 (2)	N3—C9—C10—C6	−1.2 (4)
O3—C1—C3—C4	−3.0 (4)	C7—C6—C10—C9	−1.8 (3)
C5—N2—C4—C3	0.6 (2)	C5—C6—C10—C9	176.7 (2)
C5—N2—C4—C2	−175.55 (19)		

Symmetry codes: (i) −*x*+1/2, −*y*+1/2, −*z*+1.

### Hydrogen-bond geometry (Å, °)

**Table:**

*D*—H···*A*	*D*—H	H···*A*	*D*···*A*	*D*—H···*A*
O1—H1B···N2^ii^	0.82 (3)	2.08 (3)	2.898 (3)	178 (3)
N3—H3···O5^iii^	0.88	1.89	2.755 (2)	169.
O4—H4A···O3	0.89 (2)	1.58 (2)	2.459 (2)	173 (3)

Symmetry codes: (ii) −*x*−1/2, −*y*+1/2, −*z*+1; (iii) −*x*−1/2, *y*−1/2, −*z*+3/2.
